# Remote cognitive-motor dual-task training and cognition in mild cognitive impairment: a randomized controlled trial

**DOI:** 10.1038/s41598-026-56161-w

**Published:** 2026-06-06

**Authors:** Jing Cui, Li Song, Jing wen Du, Zhiming Bian, Xiaoyi Wang, Weijun Gong

**Affiliations:** 1 Beijing Rehabilitation Hospital, Capital Medical University, Beijing, 100144 China; 2 Beijing Rehabilitation Medicine Academy, Capital Medical University, Beijing, 100069 China; 3 Department of Rehabilitation, Binzhou Medical University Hospital, Shandong Province, Bin Zhou, 256600 China; 4 Beijing Wispirit Technology Co. Ltd., Beijing, 100120 China; 5 Department of Neurorehabilitation, Beijing Rehabilitation Hospital, Capital Medical University, Beijing, 100144 China

**Keywords:** Mild cognitive impairment, remote cognitive-motor dual-task training, functional near-infrared spectroscopy, Diseases, Medical research, Neurology, Neuroscience

## Abstract

The rising prevalence of Mild Cognitive Impairment (MCI) demands effective early interventions to delay progression to dementia. This randomized controlled trial evaluated the effects of remote cognitive-motor dual-task training on cognitive function and brain functional connectivity in older adults with MCI. Linear mixed-effects models (subjects as random effects; group, time, and their interaction as fixed effects) revealed significant Group × Time interactions for MoCA scores, Mini-Mental State Examination (MMSE) scores, and brain functional connectivity (FC) (all *P* < 0.001), indicating that intervention effects differed across groups over time. Post-hoc comparisons showed that the Remote Cognitive-Motor Group (RCMG) achieved significant improvements in both MoCA and MMSE scores (both *P* < 0.001), and these gains significantly exceeded those of the Control Group (CG) (*P* < 0.001). The Remote Cognitive Group (RCG) showed a significant improvement in MoCA (*P* < 0.001), whereas no significant improvement was observed in MMSE (*P* = 0.188). For brain FC, the RCMG showed significantly greater post-intervention enhancement than the CG (*P* < 0.001). Although the RCG showed a nominally significant interaction effect (*P* = 0.016), this did not remain significant after correction for multiple comparisons, and no significant difference was observed between RCMG and RCG. Region-of-interest (ROI) analyses revealed that the RCMG exhibited significantly enhanced FC between multiple prefrontal and motor-related regions, including the mPFC, DLPFC, and PMC (*P* < 0.05).

**Trial registration** Study on rehabilitation training of cognitive-motor dual tasks for aging-related cognitive decline (ChiCTR2200064684) and the registration date was 10/14/2022.

## Introduction

The rapid growth of the global aging population poses mounting public health challenges. By 2050, adults aged 65 and older are projected to number 1.6 billion, nearly one-sixth of the world’s population^1^. This demographic shift has coincided with a sharp rise in age-related neurodegenerative disease, with mild cognitive impairment (MCI) attracting particular attention as a precursor to dementia. Characterized by progressive decline across cognitive domains such as memory and executive function^2^, MCI affects an estimated 5–20% of adults over 653, and roughly half of those diagnosed will convert to irreversible dementia within^3–5^ years4. This transition erodes functional independence and imposes a substantial socioeconomic and healthcare burden^5^.

Pharmacological approaches, including cholinesterase inhibitors, offer temporary symptom relief but rarely produce clinically meaningful outcomes, and adverse effects drive discontinuation in up to 45% of patients^6^. Non-pharmacological interventions are consequently gaining traction: regular physical exercise has been shown to slow cognitive decline, support neurogenesis, and enhance brain function in older adults with (MCI)^7^. Cognitive training confers certain benefits^8^, yet unimodal approaches yield limited transfer to broader cognitive function and modest long-term gains^9^. Combined interventions, by contrast, are more effective at improving cognition in individuals with cognitive impairment, as supported by recent systematic reviews^10,11^. Dual-task paradigms, integrating cognitive stimulation with physical activity, outperform single-task training by promoting synergistic activation and neural integration across brain regions^12^. Mechanistically, exercise drives synaptic plasticity and neurogenesis, while concurrent cognitive training guides the incorporation of newly generated neurons into existing networks^13–16^, providing a plausible neural basis for cognitive-motor dual-task interventions^17,18^.

Despite the demonstrated efficacy of traditional rehabilitation programs, low engagement, poor attendance, and inadequate adherence remain persistent obstacles^19–23^, compounded by barriers such as transportation difficulties, deteriorating health, and disruption to daily routines^24–29^. Telerehabilitation has been shown to improve quality of life in this population^30,31^, yet randomized controlled evidence for remote cognitive-motor dual-task interventions in MCI remains scarce.

Remote Dual-Task Training (RDTT) integrates cognitive and motor tasks through a digital platform, enabling participants to perform cognitive exercises concurrently with physical activity^32^, thereby simultaneously engaging cognitive-motor neural networks and potentially producing synergistic effects.

MCI is associated with disrupted connectivity across key networks, including the Default Mode Network (DMN), the visual network, and the Sensorimotor Network (SMN)^33^. Under high cognitive load, DMN inhibitory function becomes compromised and its balance with attention and executive control networks breaks down—a central mechanism of cognitive decline^34,35^. Restoring network integration is therefore a critical therapeutic target. The Dorsolateral Prefrontal Cortex (DLPFC) serves as the core hub for multitasking; its coupling with the Primary Motor Cortex (M1) underpins cognitive-motor coordination, and prior rTMS and fMRI studies confirm that strengthening DLPFC–motor cortex coupling improves cognitive-motor integration^36–41^. Functional Near-Infrared Spectroscopy (fNIRS), non-invasive, motion-tolerant, and capable of real-time monitoring, is well-suited to cognitive-motor research^42–47^; by tracking oxyhemoglobin and deoxyhemoglobin dynamics, it enables assessment of both brain activation and network coupling, making it a valuable tool for elucidating the neural mechanisms of RDTT.

This randomized controlled trial aimed to evaluate the effects of RDTT on cognitive function in older adults with MCI, and to investigate the underlying neural mechanisms through fNIRS-based assessment of functional brain connectivity.

## Results

A total of 90 participants with complete data were included in the final analysis, all of whom completed assessments at both baseline and 12 weeks post-intervention.

### Baseline characteristics

**Fig. 1:**
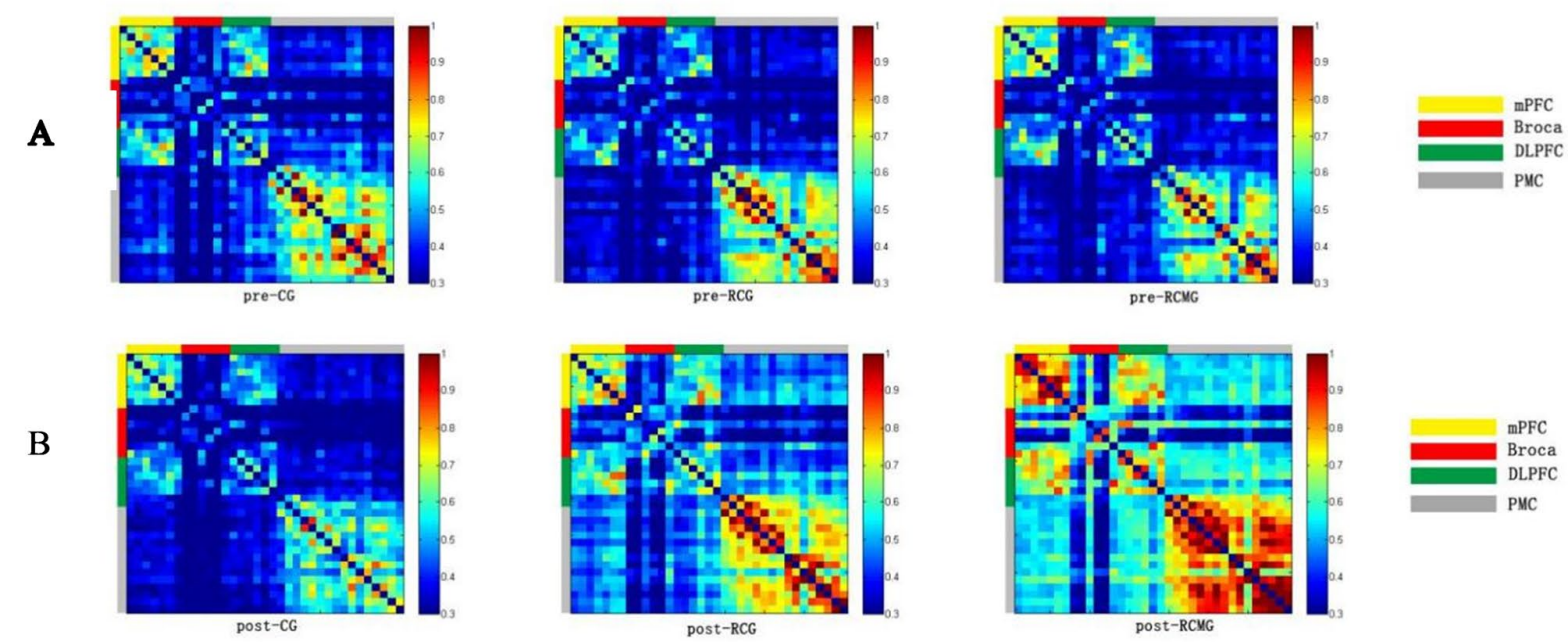
Average functional connectivity strength before and after intervention across groups. (**A**) Baseline; (**B**) Post-intervention. mPFC: medial prefrontal cortex; Broca: Broca’s area; DLPFC: dorsolateral prefrontal cortex; PMC: premotor cortex. Pre-/Post- prefixes denote timepoint for CG (control group), RCG (remote cognitive training group), and RCMG (remote cognitive-motor dual-task group). The color bar ranges from 0.3 to 1, with higher values indicating stronger connectivity. Yellow lines: mPFC connections; red lines: Broca’s area; green lines: DLPFC; gray lines: PMC.

No statistically significant differences were found among the three groups in gender, age, educational level, or health status (all *P* > 0.05), confirming baseline comparability (Table 1). Similarly, groups did not differ significantly at baseline in cognitive performance (MMSE, MoCA) or functional connectivity (FC) (Table 2) (Fig. 1).

**Table 1:** Baseline characteristics of the participants.

Variable	Category	RCMG	RCG	CG	χ² / F	*P*
Gender	Male	19 (63.3)	16 (53.3)	20 (66.7)	1.216	0.545
	Female	11 (36.7)	14 (46.7)	10 (33.3)		
Smoking status	No	12 (43.3)	15 (46.7)	17 (56.7)	1.690	0.430
	Yes	18 (56.7)	15 (53.3)	13 (43.3)		
Alcohol consumption	No	13 (43.3)	11 (36.7)	12 (40.0)	0.278	0.870
	Yes	17 (56.7)	19 (63.3)	18 (60.0)		
Self-rated health	Unhealth	21 (70.0)	19 (63.3)	22 (73.3)	0.726	0.696
	Health	9 (30.0)	11 (36.7)	8 (26.7)		
Age		69.47 ± 3.13	69.57 ± 2.93	68.17 ± 2.83	2.081	0.131
Education (years)		5.63 ± 1.71	6.03 ± 2.46	5.53 ± 2.42	0.425	0.655

Values are presented as n (%) for categorical variables and mean ± SD for continuous variables; Self-rated health was dichotomized as healthy (excellent/good/fair) and unhealthy (poor/very poor)52;Education: years of formal education; RCMG = remote cognitive–motor dual-task group; RCG = remote cognitive training group; CG = Control group.

**Table 2:** Descriptive statistics of MMSE, MoCA, and FC (mean ± SD).

Group	MMSE (Pre)	MMSE (Post)	MoCA (Pre)	MoCA (Post)	FC (Pre)	FC (Post)
RCMG	22.97 ± 3.46	27.70 ± 1.02	16.87 ± 4.64	23.47 ± 4.22	0.39 ± 0.13	0.54 ± 0.15
RCG	24.93 ± 3.68	25.97 ± 3.09	18.60 ± 4.30	21.27 ± 4.32	0.38 ± 0.13	0.46 ± 0.13
CG	23.30 ± 4.50	23.77 ± 4.01	18.07 ± 4.19	18.13 ± 4.14	0.38 ± 0.13	0.35 ± 0.12

Data are presented as mean ± standard deviation (SD). RCMG = remote cognitive–motor dual–task group; RCG = remote cognitive training group; CG = control group, Pre: baseline; Post: after intervention.

### Primary Outcome—Montreal Cognitive Assessment (MoCA)

The linear mixed-effects model identified a significant Group × Time interaction for MoCA scores (χ²(2) = 47.267, *P* < 0.001). Compared with the CG, the Time × Group interaction terms were significant for RCMG (β = 6.533, 95% CI 4.898–8.169, *P* < 0.001) and RCG (β = 2.600, 95% CI 0.965–4.235, *P* = 0.002) (Table 3).

**Table 3:** Results of linear mixed-effects models for MMSE, MoCA and FC.

Outcome	Effect	β	SE	Test statistic	95% CI	*P*
MMSE	Group × Time	–	–	χ²= 3 7.547	–	< 0.001
	Time (post vs. pre)	0.467	0.480	0.972	-0.474 to 1.408	0.331
	RCG vs. CG	1.633	0.882	1.852	-0.095 to 3.362	0.064
	RCMG vs. CG	-0.333	0.882	-0.378	-2.062 to 1.395	0.705
	Time × RCG	0.567	0.679	0.835	-0.764 to 1.897	0.404
	Time × RCMG	4.267	0.679	6.284	2.936 to 5.597	< 0.001
MoCA	Group × Time	–	–	χ²=47.267	–	< 0.001
	Time (post vs. pre)	0.067	0.590	0.113	–1.090 to 1.223	0.910
	RCG vs. CG	0.533	1.093	0.488	–1.609 to 2.676	0.626
	RCMG vs. CG	–1.200	1.093	–1.098	–3.342 to 0.942	0.272
	Time × RCG	2.600	0.834	3.116	0.965 to 4.235	0.002
	Time × RCMG	6.533	0.834	7.831	4.898 to 8.169	< 0.001
FC	Group × Time	–	–	χ²=16.520	–	< 0.001
	Time (post vs. pre)	–0.036	0.033	–1.092	–0.100 to 0.028	0.275
	RCG vs. CG	0.004	0.033	0.129	–0.061 to 0.070	0.897
	RCMG vs. CG	0.002	0.033	0.060	–0.064 to 0.068	0.952
	Time × RCG	0.111	0.046	2.403	0.020 to 0.202	0.016
	Time × RCMG	0.195	0.046	4.222	0.104 to 0.286	< 0.001

Linear mixed-effects models were fitted with subject as a random intercept. Group, time, and their interaction were included as fixed effects. The control group (CG) and baseline (pre) were used as reference categories. Wald z-statistics are reported for fixed effects. Group × Time interactions were evaluated using Wald χ² tests comparing nested models.

Random-effects estimates indicated substantial between-subject variability in MoCA scores, with a random intercept variance of 12.698 (SD = 3.563) and a residual variance of 5.221 (Table 4).

**Table 4:** Estimates of random effects for MMSE, MoCA, and FC.

Outcome	Random effect (Subject intercept) Variance	SD	Residual Variance (σ²)
MMSE	8.208	2.866	3.457
MoCA	12.698	3.563	5.221
FC	0.051	0.225	~ 0.010

Random effects represent between-subject variability (random intercept). Residual variance (σ²) reflects within-subject variability. SD is the square root of the variance.

Bonferroni-corrected post hoc comparisons showed that improvements in RCMG exceeded those in both the CG (Δ = 6.533, *P* < 0.001) and the RCG (Δ = 3.933, *P* < 0.001), and that RCG also showed greater improvement than the CG (Δ = 2.600, *P* = 0.011) (Table 5).

**Table 5:** Post hoc comparisons (Bonferroni corrected).

Outcome	Comparison	β	SE	Test statistic	95% CI	*P*
MMSE	CG (post–pre)	0.467	0.480	0.972	-0.474 to 1.408	1.000
	RCG (post–pre)	1.033	0.480	2.152	0.092 to 1.974	0.188
	RCMG (post–pre)	4.733	0.480	9.860	3.792 to 5.674	< 0.001
	ΔRCG − ΔCG	0.567	0.679	0.835	-0.764 to 1.897	1.000
	ΔRCMG − ΔCG	4.267	0.679	6.284	2.936 to 5.597	< 0.001
	ΔRCMG − ΔRCG	3.700	0.679	5.449	2.369 to 5.031	< 0.001
MoCA	CG (post–pre)	0.067	0.590	0.113	–1.090 to 1.223	1.000
	RCG (post–pre)	2.667	0.590	4.520	1.510 to 3.823	< 0.001
	RCMG (post–pre)	6.600	0.590	11.187	5.444 to 7.756	< 0.001
	ΔRCG − ΔCG	2.600	0.834	3.116	0.965 to 4.235	0.011
	ΔRCMG − ΔCG	6.533	0.834	7.831	4.898 to 8.169	< 0.001
	ΔRCMG − ΔRCG	3.933	0.834	4.716	2.298 to 5.569	< 0.001
FC	CG (post–pre)	–0.036	0.033	–1.092	–0.100 to 0.028	1.000
	RCG (post–pre)	0.075	0.033	2.307	0.011 to 0.139	0.126
	RCMG (post–pre)	0.159	0.033	4.881	0.095 to 0.223	< 0.001
	ΔRCG − ΔCG	0.111	0.046	2.403	0.020 to 0.202	0.098
	ΔRCMG − ΔCG	0.195	0.046	4.222	0.104 to 0.286	< 0.001
	ΔRCMG − ΔRCG	0.084	0.046	1.819	–0.007 to 0.175	0.414

Post hoc comparisons were performed using linear contrasts derived from the mixed-effects model. P values were adjusted using the Bonferroni correction. Δ represents the between-group difference in change (post–pre), corresponding to the estimated interaction effect (β) from the mixed-effects model.

### Secondary Outcome—Mini-Mental State Examination (MMSE)

A significant Group × Time interaction was observed for MMSE scores (χ²(2) = 37.547, *P* < 0.001). The Time × Group interaction was significant for RCMG (β = 4.267, 95% CI 2.936–5.597, *P* < 0.001), but not for RCG (*P* = 0.404) (Table 3).

Random-effects analysis indicated substantial between-subject variability in MMSE scores, with a random intercept variance of 8.208 (SD = 2.866) and a residual variance of 3.457 (Table 4).

Post hoc analyses showed that improvement in RCMG was significantly greater than in both the CG (Δ = 4.27, *P* < 0.001) and the RCG (Δ = 3.70, *P* < 0.001), despite the absence of a significant within-group change in RCG (Table 5).

### Brain Functional Connectivity (fNIRS)

The linear mixed-effects model revealed a significant Group × Time interaction for FC (χ²(2) = 16.520, *P* < 0.001). The Time × Group interaction was significant for RCMG (β = 0.195, *P* < 0.001) and reached nominal significance for RCG (β = 0.111, *P* = 0.016) (Table 3).

However after Bonferroni correction, the within-group change in RCG was not statistically significant (*P* = 0.126). Post hoc comparisons showed that RCMG exhibited significantly greater FC change than the CG (Δ = 0.195, *P* < 0.001), whereas neither the RCG vs. CG (*P* = 0.098) nor the RCMG vs. RCG (*P* = 0.414) comparisons reached statistical significance (Table 5).

### ROI analysis

**Fig. 2:**
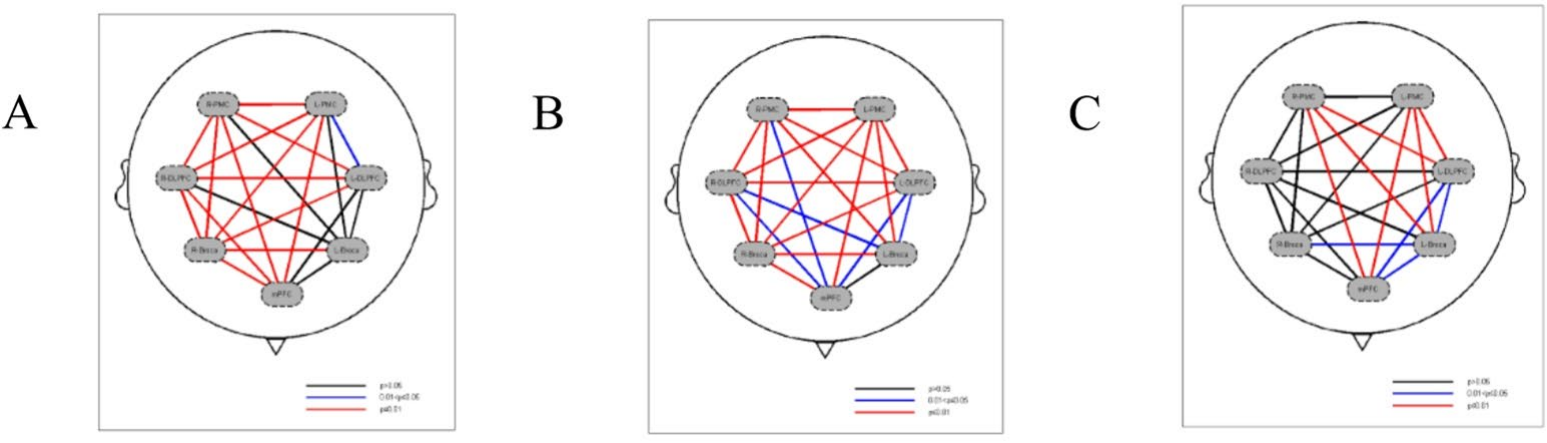
ROI-to-ROI functional connectivity before and after intervention in the RCMG, RCG, and CG groups. (**A**) RCMG; (**B**) RCG; (**C**) CG. RDLPFC/LDLPFC: right/left dorsolateral prefrontal cortex; mPFC: medial prefrontal cortex; LPMC/RPMC: left/right premotor cortex; RBroca: right-hemisphere homolog of Broca’s area; LBroca: left Broca’s area. Blue lines: *P* < 0.05; red lines: *P* < 0.01; black lines: non-significant (*P* > 0.05). The circle represents the head; the triangle indicates the nose. ROI: region of interest.

ROI analysis demonstrated significantly enhanced FC between prefrontal and motor-related regions in the RCMG (*P* < 0.05) (Table 6; Fig. 2).

**Table 6:** Comparison of ROI-to-ROI functional connectivity strength among groups after intervention.

ROIs	RCMG	RCG	CG	*P*-value	F
m PFC − R PMC	0.59	0.50	0.38	0.012*	4.81
mPFC − L PMC	0.61	0.51	0.37	0.005**	6.30
mPFC − R Broca	0.57	0.42	0.32	< 0.001**	11.99
mPFC − R DLPFC	0.81	0.62	0.57	0.012*	4.82
mPFC − L DLPFC	0.77	0.69	0.55	0.013*	4.68
R DLPFC − R PMC	0.63	0.46	0.46	0.008**	5.38
R DLPFC − L PMC	0.63	0.44	0.37	0.004**	6.74
R DLPFC − L Broca	0.44	0.39	0.29	0.045*	3.26
R DLPFC − L DLPFC	0.78	0.60	0.52	0.007**	5.56
L DLPFC − R PMC	0.68	0.54	0.38	0.005**	6.15
L DLPFC − L PMC	0.63	0.55	0.39	0.005**	6.37
L DLPFC − L Broca	0.54	0.52	0.39	0.014*	4.54
R Broca − L PMC	0.47	0.36	0.26	< 0.001**	10.67
L Broca − L PMC	0.44	0.39	0.24	0.001**	8.13
R Broca − R DLPFC	0.62	0.48	0.32	< 0.001**	11.10
R Broca − L DLPFC	0.54	0.42	0.29	< 0.001**	9.68
R Broca − L Broca	0.58	0.50	0.36	< 0.001**	10.86
R PMC − L PMC	0.91	0.75	0.65	0.003**	7.31
R PMC − R Broca	0.53	0.37	0.23	< 0.001**	9.21
R PMC − L Broca	0.37	0.36	0.22	0.006**	5.93

RCMG = remote cognitive-motor dual-task group; RCG = remote cognitive training group; CG = control group; ROI = region of interest; mPFC = medial prefrontal cortex; DLPFC = dorsolateral prefrontal cortex; PMC = premotor cortex; Broca = Broca’s area. *P < 0.05; **P < 0.01.

## Discussion

The findings suggest that RDTT is associated with improvements in cognitive function and brain functional connectivity in older adults with MCI. The combination of cognitive and physical training may confer synergistic benefits, with ROI analyses pointing to strengthened connectivity among the DLPFC, mPFC, and motor-related regions, particularly the PMC, implicating the prefrontal-motor network in MCI intervention, though these exploratory findings require further validation.

Both the RCMG and RCG improved cognitive performance, with the RCMG achieving greater gains that reached significance across multiple between-group comparisons. MMSE trends largely mirrored those of the MoCA, corroborating the primary findings. Post-hoc Bonferroni comparisons confirmed that the RCMG improved significantly on both MoCA and MMSE relative to the CG, whereas the RCG reached significance only on the MoCA; its MMSE gains did not differ significantly from the CG. These results suggest RDTT may carry a cognitive advantage over cognitive training alone, though its incremental benefit warrants confirmation in larger samples.

The greater magnitude of RCMG improvement may reflect the simultaneous engagement of multiple functional systems^48,49^. By embedding cognitive processing and motor execution within a unified task framework, RDTT more closely replicates the complexity of real-world demands than purely cognitive training. Participants must concurrently manage attention allocation, working memory, executive control, and motor coordination, a degree of multisystem activation that may foster cognitive integration and functional compensation. Moderate physical activity may also provide a more favorable neurophysiological substrate for cognition by improving cerebral perfusion, upregulating neurotrophic factors, and enhancing neuroplasticity^50^.

The prefrontal cortex, particularly the DLPFC and mPFC, is central to executive control, attention, working memory, and task switching^51^, while the PMC governs motor preparation and action planning. Dual-task conditions demand heightened prefrontal resource recruitment and task coordination^52^, and under high cognitive load the mPFC may be particularly engaged in motivational maintenance, goal-directed regulation, and task monitoring. The increased mPFC–DLPFC and mPFC–PMC connectivity observed in the RCMG may therefore reflect enhanced engagement of prefrontal networks subserving attentional control, task switching, and motor preparation during remote dual-task training. As these findings stem from exploratory ROI analyses, they should be interpreted cautiously and require independent replication.

To contextualize these findings and assess potential confounds, we examined regression to the mean and assessor bias. Baseline RCMG MoCA scores fell within the typical MCI range without extreme values, and a standardized assessment protocol was used throughout. Given that the CG showed no comparable improvement, and that fNIRS revealed concurrent FC changes, it is unlikely that regression to the mean explains the cognitive gains. Assessor bias was addressed through a single-blind design in which the assessment and intervention teams were separated, and assessors received standardized training, though residual influence cannot be entirely excluded. Baseline educational level was comparable across groups (*P* > 0.05), and the convergent MoCA and MMSE trends further argue against educational differences as a confounding explanation. Taken together, the findings likely reflect genuine intervention effects rather than methodological artifacts.

## Limitations and future directions

Several limitations warrant consideration. Practice effects are a recognized concern with repeated cognitive assessment. Although training tasks were designed to minimize content overlap with outcome measures, participants may nonetheless improve as they become more familiar with test formats. Prior work has shown that instruments such as the MoCA and MMSE are susceptible to practice effects on repeated administration^53^, particularly over shorter follow-up intervals^54^. The absence of significant improvement in the CG partly mitigates this concern, but a contribution of practice effects cannot be ruled out. Future work should employ longer intervention periods, parallel scale versions, or a broader array of objective neurophysiological measures to disentangle genuine treatment effects from familiarity-driven gains. Beyond this, the relatively modest sample size and the spatial resolution constraints inherent to fNIRS limit the strength of conclusions regarding neural mechanisms. Multimodal neuroimaging, larger multicenter designs, and extended follow-up are needed to establish the durability and generalizability of these effects. The MoCA, while widely used, has limited subdomain sensitivity; incorporating comprehensive neuropsychological batteries in future studies would improve precision. Finally, the optimal RDTT dosage and its suitability across diverse clinical populations remain open questions.

## Conclusion

RDTT is associated with cognitive improvement and enhanced brain functional connectivity in older adults with MCI. Specifically, the intervention may strengthen connectivity among the DLPFC, mPFC, and PMC, potentially reflecting improved coordination between cognitive control and motor control networks—though the precise mechanisms remain to be established. As a remotely delivered intervention, RDTT demonstrates feasibility and clinical promise; however, its long-term efficacy and broader applicability must be confirmed in larger, longer-term trials.

## Methods

### Study design

This single-center, parallel-group randomized controlled trial was registered on October 14, 2022, and comprised three arms: the Remote Cognitive-Motor Dual-Task Group (RCMG), the Remote Cognitive Group (RCG), and the Control Group (CG). Protocol modifications did not affect the primary objectives or statistical analysis plan.

Participants were eligible if they: (1) met diagnostic criteria for MCI per the Expert Consensus on Neuropsychological Assessment of Mild Cognitive Impairment, which aligns with the Petersen and NIA-AA criteria and requires subjective cognitive decline, objective cognitive impairment, preserved basic activities of daily living, and absence of dementia; (2) had an MMSE score within the education-adjusted normal range and a MoCA score < 26; (3) had no visual or auditory impairment sufficient to preclude remote participation; (4) had no history of neurological or systemic conditions known to affect cognition; (5) were aged ≥ 65 years; and (6) provided informed consent alongside a family member. Exclusion criteria were: physical disability, severe osteoporosis, or prior brain surgery; participation in any systematic rehabilitation program within the preceding 12 months; a diagnosis of Alzheimer’s disease or other dementia; cognitive impairment attributable to psychiatric disorders or substance abuse; and current use of medications with significant adverse cognitive effects (e.g., anticholinergics, sedative-hypnotics).

Recruitment ran from June 2023 to December 2024. All eligible consenting participants enrolled in a 12-week intervention, with MMSE, MoCA, and fNIRS assessments conducted at baseline and at 12 weeks.

### Sample size

Sample size was estimated using G*Power 3.1. For a three-group design with a conservative effect size of f = 0.20, α = 0.05, and power of 1 − β = 0.90, the minimum required sample was 84 participants. Accounting for an anticipated 20% dropout, the enrollment target was set at 105. A total of 107 participants were recruited; 90 completed all interventions and assessments and were included in the final analysis.

### Randomization and blinding

Group allocation was performed using a random number table, with the randomization scheme generated and sealed by an independent statistician; all analyses were conducted after study completion. Assessors were blinded to group assignments and intervention protocols, and received standardized training prior to conducting both cognitive assessments and fNIRS data acquisition. Participants were aware of their assigned intervention.

### Instruments

**Fig. 3:**
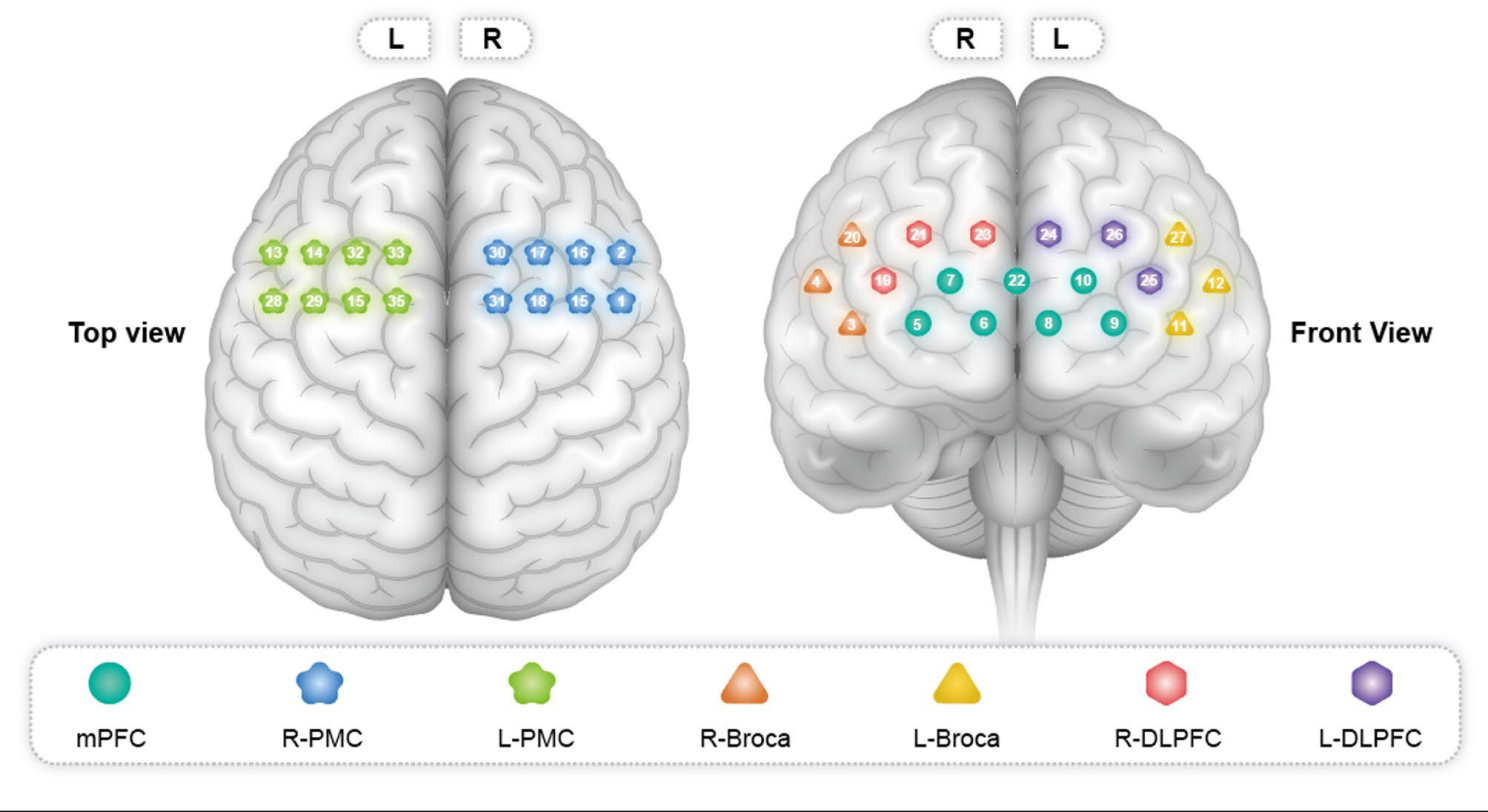
fNIRS probe configuration and channel layout. Thirty-five channels shown in top (**A**) and front (**B**) views. R: right; L: left; m PFC: medial prefrontal cortex; RPMC/LPMC: right/left premotor cortex; R Broca/L Broca: right-hemisphere homolog/left Broca’s area; RDLPFC/LDLPFC: right/left dorsolateral prefrontal cortex. Colors and shapes denote distinct regions of interest.

Cognitive training was delivered through the 66 Brain Cognitive Digital Therapy Intelligent Platform (V3.0, Beijing Zhi jing ling Technology Co., Ltd., China), which supports adaptive task adjustment and remote monitoring. Motor training was performed on a resistance-adjustable rehabilitation ergometer (model GD986, Shanghai Fanda Sports Rehabilitation Equipment Co., Ltd.), synchronized with the cognitive platform at a pedaling cadence of 50–60 rpm. Hemodynamic data were acquired using an fNIRS system (NirSmart-3000AS; Danyang Hui chuang Medical Equipment Co., Ltd., China) operating at wavelengths of 760/850 nm and a sampling rate of 10 Hz, with a 35-channel probe cap covering the prefrontal and parietal cortices (Fig. 3).

### Interventions

The RCMG and RCG each trained three times per week for 12 weeks (30–40 min per session), under equivalent supervisory conditions. All sessions were monitored remotely in real time by two trained therapists—one overseeing cognitive guidance and one ensuring safety and adherence—and participants received weekly follow-up calls or video consultations.

### RCMG group

RCMG sessions began with a 5-minute warm-up (calisthenics, marching in place, and joint mobility exercises), followed by 30 min of simultaneous cognitive training on the platform and cycling on the ergometer. Tasks targeted attention, memory, executive function, and language (e.g., shape matching, facial recognition), with difficulty adjusted in real time based on accuracy and response speed; all data were automatically logged and remotely monitored via a backend system. Sessions concluded with 5–10 min of stretching and deep breathing.

### RCG group

RCG participants completed the same 30-minute cognitive training tasks independently on the platform, targeting identical cognitive domains, with no motor training component. For both intervention groups, real-time backend monitoring and weekly follow-up contacts ensured protocol adherence. CG participants maintained their usual daily activities only; researchers conducted weekly 10–15-minute calls focused solely on health status monitoring, with no therapeutic content.

### Adherence and adverse events

Of 107 enrolled participants, 90 completed all interventions and assessments (dropout rate: 15.8%). Adherence in the intervention groups was high: RCMG and RCG participants completed an average of 33.5 ± 5.5 sessions, with a mean task completion rate of 83.4 ± 2.8% and cumulative training time of 1,068 ± 102 min per participant. No protocol deviations or serious adverse events occurred. A small number of participants reported mild, transient discomfort (muscle soreness, fatigue), all of which resolved spontaneously without medical intervention.

### Outcome measures

Assessments were conducted under standardized conditions, a uniform testing environment, standardized procedures, trained, blinded assessors, with results withheld from participants. Culturally validated instruments were used throughout: the MoCA (Beijing Version) as the primary outcome measure, and the Chinese MMSE as a secondary cognitive measure. An unauthorized version of the Chinese MMSE was used by the study team without permission, however this has now been rectified with PAR. The MMSE is a copyrighted instrument and may not be used or reproduced in whole or in part, in any form or language, or by any means without written permission of PAR (www.parinc.com). fNIRS-derived functional brain connectivity, quantified as connectivity strength between predefined ROIs, served as an additional secondary outcome. The inter-assessment interval was 12 weeks.

### Statistical analysis

All analyses were performed in SPSS 27.0. Quantitative data are reported as mean ± SD. Baseline characteristics were compared across groups using one-way ANOVA or chi-square tests. Intervention effects on MoCA scores, MMSE scores, and FC were evaluated using linear mixed-effects models (LMM), which accommodate within-subject correlations in repeated-measures data without requiring sphericity. Participants were modeled as random effects; group (RCMG, RCG, CG), time (baseline vs. post-intervention), and their interaction were modeled as fixed effects, with the CG and baseline serving as reference levels. Linear mixed-effects models were fitted using restricted maximum likelihood (REML) estimation.The Group × Time interaction was tested via likelihood-ratio test; where significant, model-based post-hoc comparisons examined within- and between-group changes, with Bonferroni correction applied for multiple comparisons. Effect sizes are reported as model estimates (β) with 95% confidence intervals. All tests were two-sided, with *P* < 0.05 considered statistically significant.

## Data Availability

All data have been included in this manuscript.

## References

[1] World Health Organization. Ageing and health. Fact sheet. (2022). Available from: https://www.who.int/news-room/fact-sheets/detail/ageing-and-health

[2] Albert, M. et al. The diagnosis is of mild cognitive impairment due to Alzheimer’s disease: recommendations from the National Institute on Aging-Alzheimer’s Association work groups on diagnostic guidelines for Alzheimer’s disease. *Alzheimers Dement.* **7** (3), 270–279 (2011). 21514249 10.1016/j.jalz.2011.03.008 PMC3312027

[3] Hu, C. et al. The prevalence and progression of mild cognitive impairment among clinic and community populations: A systematic review and meta-analysis. *Int. Psychogeriatr.* **29** (10), 1595–1608 (2017). 28884657 10.1017/S1041610217000473

[4] Petersen, R. et al. Practice guideline update summary: Mild cognitive impairment. *Neurology* **90** (3), 126–135 (2018). 29282327 10.1212/WNL.0000000000004826 PMC5772157

[5] Livingston, G. et al. Dementia prevention, intervention, and care, 2020 report of the Lancet Commission. *Lancet* **396** (10248), 413–446 (2020). 32738937 10.1016/S0140-6736(20)30367-6 PMC7392084

[6] Blanco-Silvente, L. et al. Discontinuation, efficacy, and safety of cholinesterase inhibitors for Alzheimer’s disease: A meta-analysis and meta-regression of 43 randomized clinical trials enrolling 16,106 patients. *Int. J. Neuropsychopharmacol.* **20** (7), 519–528 (2017). 28201726 10.1093/ijnp/pyx012 PMC5492783

[7] Chandra, L. et al. Effect of exercise on cognition, conditioning, muscle endurance, and balance in older adults with mild cognitive impairment: a randomized controlled trial. *J. Geriatr. Phys. Ther.* **42** (2), E15–E22 (2019). 29738405 10.1519/JPT.0000000000000191

[8] Ball, K. et al. Effects of cognitive training interventions with older adults: a randomized controlled trial. *JAMA* **288** (18), 2271–2281 (2002). 12425704 10.1001/jama.288.18.2271 PMC2916176

[9] Lin, X., Lan, P., Che, T. & Su, Y. Study on the Mechanism and Influence of Cognitive Training on Alzheimer’s Disease. *Hans J. Biomed*. **13** (2), 637–638 (2023).

[10] Law, L. et al. Influence of sequential vs. simultaneous dual-task exercise training on cognitive function in older adults. *Front. Aging Neurosci.* **9**, 368 (2017). 29163146 10.3389/fnagi.2017.00368 PMC5681915

[11] Karssemeijer, E. G. A. et al. Positive effects of combined cognitive and physical exercise training on cognitive function in older adults with mild cognitive impairment or dementia: A meta-analysis. *Ageing Res. Rev.* **40**, 75–83 (2017). 28912076 10.1016/j.arr.2017.09.003

[12] Zheng, Y. et al. Dual-task training to improve cognitive impairment and walking function in Parkinson’s disease patients: A brief review. *Sports Med. Health Sci.* **3** (4), 202–206 (2021). 35783369 10.1016/j.smhs.2021.10.003 PMC9219296

[13] Law, L. et al. Effects of combined cognitive and exercise interventions on cognition in older adults with and without cognitive impairment: a systematic review. *Ageing Res. Rev.* **15**, 61–75 (2014). 24632497 10.1016/j.arr.2014.02.008

[14] Bamidis, P. et al. A review of physical and cognitive interventions in aging. *Neurosci. Biobehav Rev.* **44**, 206–220 (2014). 24705268 10.1016/j.neubiorev.2014.03.019

[15] Curlik, D. & Shors, T. Training your brain: do mental and physical (MAP) training enhance cognition through the process of neurogenesis in the hippocampus? *Neuropharmacology* **64**, 506–514 (2013). 22898496 10.1016/j.neuropharm.2012.07.027 PMC3445739

[16] Fissler, P. et al. Novelty interventions to enhance broad cognitive abilities and prevent dementia: synergistic approaches for the facilitation of positive plastic change. *Prog Brain Res.* **207**, 403–434 (2013). 24309264 10.1016/B978-0-444-63327-9.00017-5

[17] Fabel, K. & Kempermann, G. Physical activity and the regulation of neurogenesis in the adult and aging brain. *Neuromolecular Med.* **10** (2), 59–66 (2008). 18286387 10.1007/s12017-008-8031-4

[18] Langdon, K. & Corbett, D. Improved working memory following novel combinations of physical and cognitive activity. *Neurorehabil Neural Repair.* **26** (5), 523–532 (2012). 22157145 10.1177/1545968311425919

[19] Vander Wardt, V. et al. Adherence support strategies for exercise interventions in people with mild cognitive impairment and dementia: A systematic review. *Prev. Med. Rep.* **7**, 38–45 (2017). 28593121 10.1016/j.pmedr.2017.05.007 PMC5447393

[20] Canevelli, M. et al. Sundowning in dementia: Clinical relevance, pathophysiological determinants, and therapeutic approaches. *Front. Med. (Lausanne)*. **3**, 73 (2016). 28083535 10.3389/fmed.2016.00073 PMC5187352

[21] Van Alphen, H. J. M., Hortobágyi, T. & van Heuvelen, M. J. G. Barriers, motivators, and facilitators of physical activity in dementia patients: a systematic review. *Arch. Gerontol. Geriatr.* **66**, 109–118 (2016). 27295140 10.1016/j.archger.2016.05.008

[22] Spruit, M. A. et al. An official American Thoracic Society/European Respiratory Society statement: key concepts and advances in pulmonary rehabilitation. *Am. J. Respir Crit. Care Med.* **188**, e13–e64 (2013). 24127811 10.1164/rccm.201309-1634ST

[23] Ng, L. W. et al. Does exercise training change physical activity in people with COPD? A systematic review and meta-analysis. *Chron. Respir Dis.* **9**, 17–26 (2012). 22194629 10.1177/1479972311430335

[24] Keating, A., Lee, A. L. & Holland, A. E. What prevents people with chronic obstructive pulmonary disease from attending pulmonary rehabilitation? A systematic review. *Chron. Respir Dis.* **8**, 89–99 (2011). 21596892 10.1177/1479972310393756

[25] Spruit, M. A. et al. Differences in content and organisational aspects of pulmonary rehabilitation programmes. *Eur. Respir J.* **43**, 1326–1337 (2014). 24337043 10.1183/09031936.00145613

[26] Fischer, M. et al. Drop-out and attendance in pulmonary rehabilitation: the role of clinical and psychosocial variables. *Respir Med.* **103**, 1564–1571 (2009). 19481919 10.1016/j.rmed.2008.11.020

[27] Milner, S. et al. Rate of, and barriers and enablers to, pulmonary rehabilitation referral in COPD: a systematic scoping review. *Respir Med.* **137**, 103–114 (2018). 29605192 10.1016/j.rmed.2018.02.021

[28] Bjoernshave, B., Korsgaard, J. & Nielsen, C. Does pulmonary rehabilitation work in clinical practice? A review on selection and dropout in randomized controlled trials on pulmonary rehabilitation. *Clin. Epidemiol.* **2**, 73–83 (2010). 20865106 10.2147/clep.s9483 PMC2943180

[29] Bjoernshave, B. et al. Participation in pulmonary rehabilitation in routine clinical practice. *Clin. Respir J.* **5**, 235–244 (2011). 21324098 10.1111/j.1752-699X.2011.00237.x

[30] Md Fadzil, N. H. et al. A scoping review for usage of telerehabilitation among older adults with mild cognitive impairment or cognitive frailty. *Aging Ment Health*. **27** (1), 1–14 (2023). 35409683 10.3390/ijerph19074000 PMC8997970

[31] Cotelli, M. et al. Cognitive telerehabilitation in mild cognitive impairment, Alzheimer’s disease and frontotemporal dementia: A systematic review. *J. Telemed Telecare*. **25** (2), 67–79 (2019). 29117794 10.1177/1357633X17740390

[32] Yıldız, H. & Demir, T. Effectiveness of motor-cognitive dual-task exercise via telerehabilitation in Alzheimer’s disease: An online pilot randomized controlled study. *Clin. Neurol. Neurosurg.* **223**, 107501 (2022). 36368169 10.1016/j.clineuro.2022.107501

[33] Hou, Y. et al. Different resting-state network disruptions in newly diagnosed drug-naïve Parkinson’s disease patients with mild cognitive impairment. *BMC Neurol.* **21**, 327 (2021). 34433445 10.1186/s12883-021-02360-z PMC8386092

[34] Sakoglu, U. Paradigm shift in translational neuroimaging of CNS disorders. *Biochem. Pharmacol.* **81**, 1374–1387 (2011). 21219879 10.1016/j.bcp.2010.12.029

[35] Menon, V. 20 years of the default mode network: A review and synthesis. *Neuron* **111** (16), 2469–2487 (2023). 37167968 10.1016/j.neuron.2023.04.023 PMC10524518

[36] Xu, B. et al. Using Dual-Target rTMS, Single-Target rTMS, or Sham rTMS on Post-Stroke Cognitive Impairment. *J. Integr. Neurosci.* **23** (8), 161 (2024). 39207080 10.31083/j.jin2308161

[37] Li, H. et al. Role of the medial prefrontal cortex in dual-task performance: An fMRI study. *Brain Cogn.* **172**, 105987 (2024).

[38] Li, Y. et al. Cerebral Functional Manipulation of Repetitive Transcranial Magnetic Stimulation in Cognitive Impairment Patients After Stroke: An fMRI Study. *Front. Neurol.* **11**, 977 (2020). 33013646 10.3389/fneur.2020.00977 PMC7506052

[39] Yin, M. et al. Effects of rTMS Treatment on Cognitive Impairment and Resting-State Brain Activity in Stroke Patients: A Randomized Clinical Trial. *Front. Neural Circuits*. **14**, 563777 (2020). 33117131 10.3389/fncir.2020.563777 PMC7561423

[40] Ding, X. et al. Patterns in default-mode network connectivity for determining outcomes in cognitive function in acute stroke patients. *Neuroscience* **277**, 637–646 (2014). 25090922 10.1016/j.neuroscience.2014.07.060

[41] Jiang, L. et al. Decreased functional connectivity within the default-mode network in acute brainstem ischemic stroke. *Eur. J. Radiol.* **105**, 221–226 (2018). 30017284 10.1016/j.ejrad.2018.06.018

[42] Ferrari, M. & Quaresima, V. A brief review on the history of human functional near-infrared spectroscopy (fNIRS) development and fields of application. *Neuroimage* **63**, 921–935 (2012). 22510258 10.1016/j.neuroimage.2012.03.049

[43] Ayaz, H. et al. Continuous monitoring of brain dynamics with functional near infrared spectroscopy as a tool for neuroergonomic research: empirical examples and a technological development. *Front. Hum. Neurosci.* **7**, 871 (2013). 24385959 10.3389/fnhum.2013.00871 PMC3866520

[44] Gawryluk, J. et al. Functional magnetic resonance imaging and functional near-infrared spectroscopy: insights from combined recording studies. *Front. Hum. Neurosci.* **11**, 419 (2017). 28867998 10.3389/fnhum.2017.00419 PMC5563305

[45] Whelan, H. et al. Functional near-infrared spectroscopy and its clinical application in the field of neuroscience: advances and future directions. *Front. Neurosci.* **14**, 724 (2020). 32742257 10.3389/fnins.2020.00724 PMC7364176

[46] Pinti, P. et al. The present and future use of functional near-infrared spectroscopy (fNIRS) for cognitive neuroscience. *Ann. N Y Acad. Sci.* **1464**, 5–29 (2020). 30085354 10.1111/nyas.13948 PMC6367070

[47] Shu, Z. et al. fNIRS-based graph frequency analysis to identify mild cognitive impairment in Parkinson’s disease. *J. Neurosci. Methods*. **402**, 110031 (2024). 38040127 10.1016/j.jneumeth.2023.110031

[48] Zhao, J. et al. Stage-specific functional networks associated with cognitive impairment in Parkinson’s disease: A pilot fNIRS study. *Front. Aging Neurosci.* **17**, 1562203 (2025). 40433510 10.3389/fnagi.2025.1562203 PMC12106520

[49] Rao, B. et al. Suboptimal states and frontoparietal network-centered incomplete compensation revealed by dynamic functional network connectivity in patients with post-stroke cognitive impairment. *Front. Aging Neurosci.* **14**, 893297 (2022). 36003999 10.3389/fnagi.2022.893297 PMC9393744

[50] Jones, D. & Graff-Radford, J. Executive dysfunction and the prefrontal cortex. *Continuum (Minneap Minn)*. **27** (6), 1586–1601 (2021). 34881727 10.1212/CON.0000000000001009

[51] Chinese Society of Neurology Dementia and Cognitive Disorders Group. Expert consensus on neuropsychological assessment for mild cognitive impairment (2025 edition). *Natl. Med. J. China*. **105** (3), 204–218 (2025). 10.3760/cma.j.cn112137-20240612-01322 39828578

[52] Zheng, Y., Li, C., Hua, Y., Zhang, J. & Wang, Y. Comparative effectiveness of different dual-task mode interventions on cognitive function in older adults with cognitive impairment or dementia: a systematic review and network meta-analysis. *Aging Clin. Exp. Res.* **37** (3), 3016–3025 (2025). 10.1007/s40520-025-03016-5 PMC12043736 40304821

[53] Practice effect of. repeated cognitive tests among older adults: associations with brain amyloid pathology and other influencing factors. *Front. Aging Neurosci.* **14**, 909614 (2022). 35875808 10.3389/fnagi.2022.909614 PMC9297730

[54] Cooley, S. A. et al. Longitudinal change in performance on the Montreal Cognitive Assessment in older adults. *Clin. Neuropsychol.* **29** (6), 824–835 (2015). 26373627 10.1080/13854046.2015.1087596 PMC4644436

